# Diagnosis and Management of Pulmonary Arterial Hypertension

**DOI:** 10.1155/2011/845864

**Published:** 2011-09-20

**Authors:** Jeanne Houtchens, Douglas Martin, James R. Klinger

**Affiliations:** Division of Pulmonary, Sleep and Critical Care Medicine, Rhode Island Hospital, Alpert Medical School of Brown University, Providence, RI 02903, USA

## Abstract

Pulmonary arterial hypertension is a rare disease, which requires a high index of suspicion to diagnose when patients initially present. Initial symptoms can be nonspecific and include complaints such as fatigue and mild dyspnea. Once the disease is suspected, echocardiography is used to estimate the pulmonary arterial (PA) pressure and to exclude secondary causes of elevated PA pressures such as left heart disease. Right heart catheterization with vasodilator challenge is critical to the proper assessment of pulmonary hemodynamics and to determine whether patients are likely to benefit from vasodilator therapy. Pathologically, the disease is characterized by deleterious remodeling of the distal pulmonary arterial and arteriolar circulation, which results in increased pulmonary vascular resistance. In the last fifteen years, medications from three different classes have been approved for the treatment of pulmonary arterial hypertension. These include the prostanoids, endothelin receptor antagonists, and phosphodiesterase-5 inhibitors.

## 1. Pathophysiology

Pulmonary arterial hypertension (PAH) is a progressive incurable disease that is characterized by extensive remodeling of the pulmonary circulation predominantly in the distal pulmonary arteries and arterioles. Proliferation of pulmonary vascular endothelial and smooth muscle cells leads to intimal and medial thickening of the pulmonary resistance vessels [1]. In some places, these changes are so severe that they result in near obliteration of the vascular lumen. These diffuse vascular changes increase resistance to blood flow through the lungs. As the disease progresses, the right ventricle becomes incapable of adequately increasing pulmonary blood flow during exercise and patients begin to notice exertional dyspnea. Eventually, the rise in pulmonary vascular resistance (PVR) leads to right ventricular failure, and cardiac output begins to fall even under resting conditions. In its final stages, patients become severely debilitated and are unable to perform nearly any activity without dyspnea or chest pain. If left untreated, most patients progress to overt right heart failure and death within 3 years of diagnosis [2].

Although PAH is a rare disease, it often strikes patients who are otherwise healthy in the middle of their life. Its devastating impact on the lives of thousands of people has led to an intense focus of research in pulmonary vascular biology over the last quarter century that has resulted in the development of numerous new therapies that have improved the prognosis considerably. Unfortunately, a cure for PAH has not yet been forthcoming and long-term survival remains poor. Even with modern medical therapy, most patient experience progression of their disease and many are referred for lung transplantation. The discovery this decade of a genetic defect that is associated with a substantial number of cases has raised the hope that a cure for PAH may eventually be found [3, 4].

The pathogenesis of PAH remains unclear. However, several important imbalances in mediators of pulmonary vascular cell growth and apoptosis have been described. Patients with PAH demonstrate a decrease in the synthesis of prostacyclin. This potent pulmonary vasodilator also has important inhibitory effects on platelet aggregation and cellular proliferation. Patients with PAH have decreased pulmonary expression of prostaglandin synthase, the major enzyme responsible for its synthesis from the arachidonic acid pathway. They also have a reduction in the circulating levels of PGI2 relative to circulating levels of thromboxane [5]. These changes cause a state of prostacyclin insufficiency in patients with PAH and form the rationale for the use of prostacyclin replacement therapy in the treatment of PAH.

Endothelin is a potent vasoconstrictor and smooth muscle mitogen secreted by the pulmonary endothelium. Immunohistochemistry studies have demonstrated increased expression of endothelin in the obliterative vascular lesions found in the lungs of patients with PAH [6]. Circulating levels of endothelin are also increased in PAH patients and correlate with disease severity [7]. Endothelin receptor antagonists were the first orally active agents approved for the treatment of PAH and work by blocking the mitogenic and vasoconstrictive effects of ET-1 on the pulmonary circulation.

In the healthy person, the pulmonary vasculature dilates in response to increased flow allowing the lung to accommodate a marked increase in blood flow during exercise without much of a rise in PA pressure. Synthesis and release of nitric oxide (NO) by endothelial nitric oxide synthase (eNOS) in the pulmonary vascular endothelium plays an important role in flow-mediated vasodilation in the pulmonary circulation. Patients with PAH have decreased pulmonary expression of eNOS and decreased levels of NO in exhaled air, raising the possibility that decreased NO synthesis contributes to the rise in PVR [8, 9]. The biologic effects of NO are mediated via binding to soluble guanylate cyclase and generation of cGMP. Several studies suggest that the activity of phosphodiesterase 5, the major enzyme responsible for the metabolism of cGMP is increased in animal studies of PH [10]. Inhibition of cGMP metabolism via PDE5 inhibitors is the most recent therapeutic strategy to be developed for the management of PAH.

## 2. Disease Classification

The current accepted definition of PAH is a mean PA pressure of greater than 25 mm Hg with a mean pulmonary capillary wedge pressure of less than 15 mm Hg and a pulmonary vascular resistance (PVR) of >3 Woods units. Elevation in peak PA pressure reported on transthoracic echocardiogram is not uncommon, but in the great majority of cases, it is not caused by PAH. A number of pathological states can elevate PA pressures, most notably chronic heart and lung diseases. During the past several decades, the World Health Organization (WHO) has proposed a new terminology to describe the various forms of pulmonary hypertension. The most recent update to this classification system was the 4th World Symposium on Pulmonary Hypertension in Dana Point, California, in 2008 [11] (Table 1).

WHO group 1 is termed PAH and is characterized by a diffuse pulmonary vasculopathy of the pulmonary arteries and arterioles that is progressive in nature and results in marked increases in the transpulmonary pressure gradient. This group is further subdivided into idiopathic PAH (IPAH, originally described as primary pulmonary hypertension) and heritable PAH. The latter is used to describe PAH associated with a number of genetic mutations that have been linked to the development of PAH [3, 4]. PAH occurs with increased frequency in patients with connective tissue disease, particularly the limited cutaneous form of scleroderma. Other diseases associated with a high incidence of PAH include portal hypertension, HIV infection, congenital left to right intracardiac shunts and the use of some anorectic drugs. PAH that occurs in patients with these diseases is referred to as associated PAH (APAH). Although the etiology of IPAH, heritable PAH, and the APAH may vary, they all have in common a diffuse vasculopathy of the pulmonary arterial circulation that is both progressive and severe, and thus are included in the same WHO group. All of the presently available therapies for pulmonary hypertension have indications that are limited to WHO group 1 and this is the form of pulmonary hypertension that will be discussed in this paper.

WHO group 2 refers to elevation of PA pressure that is caused by an increase in pulmonary venous pressure from left-sided heart disease. This type of pulmonary hypertension is termed pulmonary venous hypertension and is characterized by a nearly normal pulmonary vascular resistance (<3 Woods Units). WHO group 3 includes pulmonary hypertension that occurs with chronic lung diseases such as emphysema, interstitial lung disease, and sleep disordered breathing. PA pressure elevation is usually moderate in this group and often reflects the severity of the underlying lung disease. WHO group 4 refers to pulmonary hypertension that occurs in association with venous thromboembolic disease. The last WHO group is reserved for a variety of diseases known to be associated with pulmonary hypertension for unclear reasons such as pulmonary sarcoidosis.

In addition to the etiological grouping, the WHO also uses a grading score for functional class based on the model developed for heart failure by the New York Heart Association. Patients are designated as one of four functional classifications based on the severity of their dyspnea and physical limitations (Table 2). The WHO functional classification is used as an end point in PAH clinical trials and routinely during office visits as a subjective measure of disease progression.

## 3. Epidemiology

Idiopathic PAH is the most common form of the disease affecting 40%–50% of the patient in WHO diagnosis group 1. The estimated incidence of IPAH ranges from 7–50 per million [12, 13]. Approximately 2/3 of all PAH patients are female. PAH can occur at any age, but in a US-based registry, the peak incidence occurred during the fourth and fifth decades of life [2]. Approximately 6%–10% of patients with idiopathic PAH may have a family history of PAH and genetic screening has identified mutations in the bone morphogenic protein type II receptor in up to 25% of IPAH patients. Known exposure or abuse of certain drugs or toxins such as fenfluramine, amphetamines, cocaine, methamphetamines, and rapeseed oil have been associated with an increase risk of PAH. PAH is also seen with increased frequency in a handful of diseases as described above and in hemolytic anemias such as sickle cell disease and thalassemia. Although uncommon in developed countries, the leading cause of PAH in the world is schistosomiasis.

## 4. Clinical Presentation

**Figure 1:**
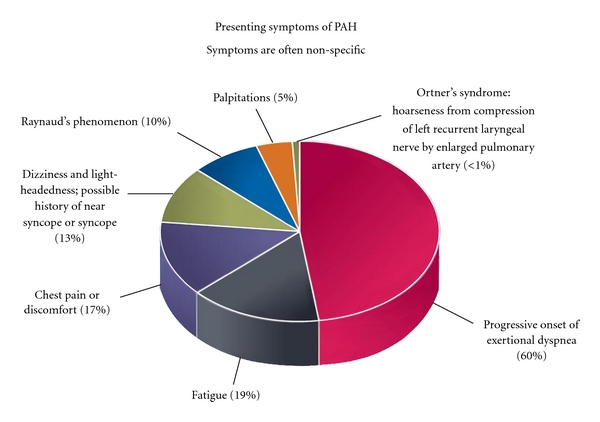


Prompt diagnosis of PAH in an early stage is important so that treatment may be started before right heart failure occurs. However, time to diagnosis once symptoms develop is typically long and is impacted by the lack of specific disease symptoms and the rarity of the disease that results in a low level of clinical suspicion. Thirty years ago, the mean time between symptom onset and diagnosis was 2 years [2]. Data from more recent registries suggest that this delay in diagnosis persists despite increased recognition and the wide spread availability of echocardiography [12, 14]. Patients with APAH may be diagnosed earlier than IPAH due to awareness of the association with certain comorbid diseases. Initial symptoms of PAH are usually subtle and nonspecific (see Figure 1). Patients often report fatigue, weakness, and shortness of breath with exertion. Typically, a patient will feel as though they are out of shape and that their fatigue is due to their lifestyle, aging, or other illnesses and at this point can still perform most activities. When symptoms progress to the point that the patient is limited with normal activities or has trouble keeping up with peers and family members, they frequently seek medical care. Delay in diagnosis typically occurs due to the broad differential diagnosis of shortness of breath and fatigue. Initial evaluation often reveals no obvious abnormality leaving the patient with a diagnosis of deconditioning or malingering. Failure to find a cause may frustrate the patient and cause them to avoid seeking additional medical care. In the absence of any identifiable pulmonary disease, most patients are eventually sent for echocardiogram that usually detects elevated PA pressure or impaired RV function. As the disease progresses, patients may complain of constant fatigue, shortness of breath and chest pain or heaviness at rest, palpitations, dizziness, peripheral edema, and exertional syncope.

When PAH is suspected from the patient's symptoms or detected by echocardiogram performed for other reasons, further evaluation should start with a complete history and physical exam. A thorough interview may reveal a family history of early death due to heart failure or other events suggestive of pulmonary hypertension. A history of other heart and lung diseases, connective tissue disease, venous thrombosis, pulmonary embolism, or hypercoagulopathy should be sought. Patients should be questioned about risk factors for HIV infection, hepatitis, or cirrhosis and asked if they are aware of any congenital or rheumatic heart disease and an assessment should be made regarding the likelihood of sleep disordered breathing. Finally, patients should be questioned about previous use of amphetamines or other appetite suppressants and travel to or immigration from countries known to be endemic for schistosomiasis.

Physical signs of elevated PA pressure are few and may be subtle. Pulmonary hypertension often results in enough tricuspid regurgitation to produce a systolic murmur best heard at the right sternal border. The second heart sound is often split due to enlargement of the right ventricle and the pulmonic component may be accentuated [15]. Signs of increased right-sided pressure such as jugular venous distension, hepatojugular reflex, and a right ventricular heave develop as the disease progresses. In advanced stages, patients develop severe lower extremity edema, ascites, and cyanosis.

The examiner should also look for signs of connective tissue diseases such as malar rash, telangiectasias, calcinosis, sclerodactyly, and Raynaud's disease. Evidence of liver cirrhosis or portal hypertension and other diseases that can contribute to the development of pulmonary hypertension such as thyroid disease, obstructive sleep apnea, and hemolytic anemia should be sought. Signs of chronic heart disease, pulmonary fibrosis, or emphysema suggest that patients may have pulmonary venous hypertension or pulmonary hypertension associated with chronic lung disease rather than PAH. 

## 5. Diagnostic Testing

Patients are most often referred for evaluation of pulmonary hypertension because of progressive dyspnea that is not readily explained by other diseases or because elevated PA pressure was found during an echocardiogram. The approach to diagnosis is similar in both scenarios. An ECG and chest film are helpful in excluding subclinical heart or lung disease and may provide additional information about the severity of the patient's PAH. Signs of elevated right-sided pressure and right heart strain such as right axis deviation, increased p-wave amplitude in lead 2 and t-wave inversion in the precordial leads suggest more severe pulmonary hypertension. Enlarged right ventricle and proximal pulmonary arteries can be seen on chest film in some patients and peripheral pruning of the pulmonary vessels may be apparent. Increased filling of the retrosternal air space on lateral chest film suggests right ventricular enlargement. Hilar adenopathy may suggest sarcoidosis.

Pulmonary function tests should be done to help exclude obstructive or restrictive lung disease and obtain a baseline measure of diffusion capacity. Adequate oxygenation should be confirmed by pulse oximetry or arterial blood gas analysis. When suspected, hypercarbia should be excluded by arterial or venous blood gas analysis. The 6 minute walk test has become a mainstay of evaluating PAH patients and the 6 minute walking distance is the most frequent primary efficacy outcome measured in clinical trials of presently available PAH therapies. The baseline 6 minute walking distance should be established ideally before initiating treatment. Change in blood pressure, pulse and oxygen saturation should be recorded along with the Borg dyspnea index. Patients who have a fall in oxygenation saturation are more likely to have pulmonary vascular disease. Inability to increase blood pressure suggests impaired cardiac function. A formal sleep study may not be necessary in all patients but should be considered in those with symptoms suggestive of obstructive sleep apnea or other forms of sleep disordered breathing.

Laboratory blood tests important in the initial evaluation of PAH include HIV test, liver function tests, thyroid function tests, and serologies to rule out lupus, scleroderma, and rheumatoid arthritis. A baseline BNP and serum sodium may be helpful in monitoring disease progression [16, 17]. Sexually active women of child-bearing age may want to have a serum pregnancy test before starting some medical therapies for PAH. A V/Q scan is necessary to exclude chronic thromboembolic disease, pulmonary veno-occlusive disease, or other disorders that may obstruct the pulmonary vasculature. A pulmonary angiogram or CT pulmonary angiogram is often used instead but is not as sensitive in detecting chronic thromboembolic disease of distal pulmonary vessels. 

A good quality transthoracic echocardiogram is extremely useful in evaluating pulmonary hypertension [18, 19]. Right ventricular systolic pressure can be calculated by measuring the speed of the regurgitant tricuspid jet using Doppler ultrasound. Right atrial pressure is estimated by looking at the degree of collapse of the inferior vena cava during inspiration. Adding the 2 together provides peak right ventricular pressure that in the absence of significant pulmonary valvular disease closely reflects peak PA pressure. Although attention is often focused on the estimated PA pressure, important information is also obtained from the evaluation of cardiac chamber sizes and function. Right atrial and or ventricular enlargement suggests chronic elevation of right sided filling pressures. Right ventricular hypokinesis is an indication of impaired right ventricular systolic function. Deviation of the intraventricular septum into the left ventricle during diastole is often described as right ventricular pressure overload or a “D” shaped intraventricular septum and indicates that right ventricular end diastolic pressure has exceeded left ventricular end diastolic pressure. Pericardial effusion is seen in later stages of PAH and is associated with a poor prognosis [20].

The echocardiogram can also help look for left-sided heart disease that may elevate PA pressure by increasing pulmonary venous pressures. Enlargement of the left atrium suggests pulmonary venous hypertension or mitral valve disease. Decreased left ventricular systolic function is readily apparent when present. Left ventricular hypertrophy raises the concern of pulmonary venous hypertension from diastolic dysfunction and some echocardiographers will report impaired diastolic filling of the left ventricle by examining the pattern of blood flow through the mitral valve during diastole. Findings such as these should alert the clinician to the possibility of left sided heart disease and consideration of additional cardiac studies.

Definitive diagnosis of PAH cannot be made without accurate measurement of PA pressure and an assessment of pulmonary venous pressure and cardiac output. At the present time, this essentially requires right heart catheterization. Right heart catheterization for the initial evaluation of PAH has become the standard of care as described by the recommendations in the Dana Point 2009 WHO consensus as well as by guidelines written and adopted by the American College of Cardiologists, American Thoracic Society, and the American College of Chest Physicians [21]. In addition, many insurance companies have adopted these guidelines when approving payment for diagnostic workup and treatment of PAH.

Some clinicians rely on echocardiogram to make the diagnosis of PAH because it is less invasive, but because of the rarity of PAH relative to that of other diseases that increase peak PA pressure, it is necessary to demonstrate that the elevation in PA pressure is due to increased PVR. This can only be done by direct catheterization of the pulmonary artery, measurement of PA pressure and downstream pulmonary venous pressure using the pulmonary artery occlusion technique, and accurate measurement of cardiac output. It is also important to realize the PA pressure is dependent on cardiac output and that a moderate elevation of PA pressure on echocardiogram can be seen in severe PAH when right ventricular failure occurs. Right heart catheterization also allows for evaluation of left to right intracardiac shunts and acute reactivity to pulmonary vasodilators. 

Medications used for vasodilator testing while the right heart catheter is in place include inhaled nitric oxide, intravenous epoprostenol, or intravenous adenosine. The definition of a positive vasodilator response is a greater than 10% decrease in pulmonary artery mean pressure to an absolute mean value of less than 40 mm/Hg with no decrease in cardiac output. In addition to acute vasodilator testing, patients with mild or moderate pulmonary hypertension are sometimes exercised with the right heart catheter in place to determine if PA pressure rises during exercise.

### 5.1. Treatment

**Figure 2:**
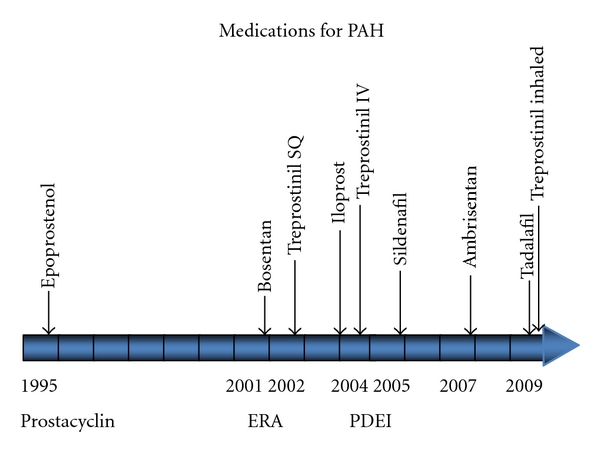


Over the last 15 years, 9 different drugs representing 3 new classes of medication have been developed for the treatment of PAH (Figure 2). This explosion in drug development was spurred by a marked increase in the understanding of pulmonary vascular biology that occurred in the last quarter of the 20th century. Prior to the approval of intravenous epoprostenol at the end of 1995, the only medications available to treat PAH were vasodilators designed to treat systemic hypertension. These agents such as hydralazine, ACE inhibitors and calcium channel blockers generally demonstrated greater vasodilatory effect on systemic than pulmonary vessels causing systemic hypotension and increasing right heart strain as the right ventricle worked to increase cardiac output to compensate for the fall in systemic vascular resistance. A small minority of patients with PAH show marked improvement in pulmonary vascular resistance and cardiac output in response to calcium channel blockers. These patients, best described by Rich et al. [22] show excellent response to long-term treatment with calcium channel blockers alone and may represent a subset of PAH patients with increased pulmonary vascular tone as opposed to pulmonary vascular remodeling. Although more recent studies [23] estimate that this group of patients represents less than 10% of all WHO group 1 PAH patients, their identification is warranted due to their much improved prognosis and successful management with calcium channel blocker agents alone. For this reason, it is highly recommended that all patients undergo acute vasodilator reactivity testing in the catheterization lab with short acting agents such as inhaled nitric oxide, intravenous epoprostenol, or adenosine as part of their initial evaluation. Those who have a positive pulmonary vasodilator response defined as a decrease in mPAP of 10 mm Hg or greater and a decrease in mPAP to below 40 mm Hg should be considered for treatment with calcium channel blockers alone. For the great majority of patients who do not respond to acute pulmonary vasodilator challenge, one of the following medical therapies described below may be considered.

#### 5.1.1. Phosphodiesterase Inhibitors

As mentioned previously, patients with pulmonary hypertension have reduced expression of endothelial nitric oxide synthase in the pulmonary vasculature [8]. While nitric oxide is a potent vasodilator, chronic administration of nitric oxide for the treatment of pulmonary hypertension is impractical. Nitric oxide's vasodilating properties are mediated through the generation of cyclic GMP (cGMP). Phosphodiesterases are a family of enzymes that hydrolize cGMP representing the major route of cGMP metabolism. The PDE-type 5 isoform is the primary PDE responsible for metabolizing cGMP in the corpus cavernosum of the penis and in the pulmonary arteries. Activity of PDE-5 may be upregulated in PAH causing a relative deficiency of cGMP in pulmonary vascular smooth muscle. PDE-5 inhibitors increase intracellular cGMP levels leading to a reduction in intracellular calcium levels and subsequent smooth muscle relaxation causing vasodilation in resistance arterioles.

Sildenafil was the first PDE5 inhibitor approved for the treatment of PAH. It had previously demonstrated potential in the treatment of pulmonary hypertension in animal models [24] and as adjunctive or sole treatment in human pulmonary hypertension [25, 26]. The largest clinical trial was a double-blind, placebo-controlled study of 278 patients from 53 centers throughout the world [27]. Patients had idiopathic PAH or PAH associated with connective tissue disease, or congenital systemic-to-pulmonary shunts. At baseline, 39% of patients were in WHO functional class II, and 58% were in class III. They were randomized to receive placebo or one of three doses of sildenafil (20, 40, or 80 mg) orally three times daily for twelve weeks. The primary endpoint was the change over 12 weeks in the 6-minute walk distance. 

Analysis of the primary endpoint demonstrated an increase in walk distance for all groups randomized to sildenafil. At week 12, the mean placebo-corrected effects were 45 m, 46 m, and 50 m for those in the 20 mg, 40 mg, and 80 mg groups respectively (*P* < 0.001 for all treatment groups compared to placebo). Analysis of hemodynamic data demonstrated a significant improvement in pulmonary vascular resistance with sildenafil, and there was a dose-response effect with the 80 mg group demonstrating the greatest reduction (−261 dyn*sec*cm^−5^ with 80 mg as compared with an increase of 49 dyn*sec*cm^−5^ in the placebo group). The improvement in cardiac index also exhibited a dose response relationship although the improvement in the 20 mg group compared with placebo did not reach statistical significance. The incidence of clinical worsening did not differ between the two treatment groups. Two hundred fifty nine of the patients enrolled in a long-term extension study of 80 mg of sildenafil three times daily. During a one year follow up, 15 of those patients withdrew, and 14 died. Of the remaining 230 patients, eight were receiving additional therapy. Analysis of the other 222 patients revealed that the mean change from baseline in the six-minute walking distance was 51 meters (95% CI of 41 to 60). 

Tadalafil is an orally administered selective PDE5 inhibitor, which has the considerable advantage of once daily dosing. Human data on the use of this compound in patients with PAH remains more limited than with sildenafil. The Pulmonary Arterial Hypertension and Response to Tadalafil (PHIRST) Study Group randomized 405 treatment-naive or bosentan treated patients with PAH to placebo or to tadalafil 2.5, 10, 20, or 40 mg orally once daily [28]. The study population included patients with idiopathic or heritable PAH, or PAH associated with connective tissue disease, HIV infection, anorexigen use, or congenital systemic-to-pulmonary shunt. Randomization was stratified for baseline walking distance, type of PAH, and bosentan use. The primary end point was the change from baseline in the 6-minute walk distance.

Tadalafil at doses of 10, 20, and 40 mg, compared with placebo, improved the six-minute walk distance in a dose-dependent manner at week 16 (mean placebo-corrected treatment effects of 14 m, 20 m, 27 m, and 33 m for the 2.5, 10, 20, and 40 mg groups, resp.). However, the 40 mg dose of tadalafil was the only one that reached the prespecified value of statistical significance of *P* < 0.01. In addition, time to clinical worsening was significantly improved in the tadalafil 40 mg group (*P* = 0.041). Fifty-three percent of the patients were on background therapy with bosentan during the trial. Bosentan-naïve patients given 40 mg of tadalafil had a greater increase in walking distance (44 meters) than patients on background bosentan (23 meters). Further supporting the idea of a blunted improvement in exercise capacity for patients already receiving PAH therapy, bosentan-naïve patients tended to have better results in all secondary end points. The improvement in 6-minute walking distance was sustained for 10 months in a subgroup of patients who had been enrolled in a long-term extension study of 20 or 40 mg tadalafil. Of the 213 patients who had completed 44 weeks of tadalafil therapy at the time of publication, the mean change in 6-minute walking distance was 37 meters at 16 weeks and 38 m after 44 weeks.

### 5.2. Prostacyclins

Prostacyclin was discovered in 1976 and was found to have vasodilating, antiplatelet, and antiproliferative properties. The chemical analogue epoprostenol was synthesized and tested in the same year [29]. The finding that patients with PAH were deficient in prostacyclin leads to a natural interest in the use of epoprostenol for therapeutic purposes. Promising results from early case reports and small series of patients with PAH treated with epoprostenol [30] as well as a small randomized trial comparing continuous epoprostenol with conventional therapy against conventional therapy alone [31] lead to a landmark multicenter randomized controlled trial that was published in 1996 [32]. That 12-week open trial enrolled 81 patients with NYHA functional class III or IV PAH. Patients were randomized to receive continuous intravenous epoprostenol plus conventional therapy or conventional therapy alone. Conventional therapy consisted of anticoagulants, calcium channel blockers, cardiac glycosides, and supplemental oxygen. Endpoints for the study were the effect of epoprostenol on exercise capacity, quality of life, hemodynamics, and survival. The epoprostenol-treated group had statistically significant better outcomes for all of these over the 12-week study period. The median 6-minute walk distance improved from 315 meters to 362 meters in the epoprostenol-treated group, while it decreased from 270 meters to 204 meters in the conventional treatment group (*P* < 0.002 for the comparison of the treatment groups). With adjustment for baseline differences in 6-minute walk distance and vasodilator use, the epoprostenol treated group still exhibited a median increase of 31 meters, while the conventional therapy group exhibited a median decrease of 29 meters (*P* < 0.02). Quality of life as measured by the Chronic Heart Failure Questionnaire improved only in the epoprostenol-treated group. The mean PA pressure decreased 4.8 ± 1.3 mm Hg in the epoprostenol treated group, while it increased 1.9 ± 1.6 mm Hg in the conventional therapy group (difference between treatment groups of −6.7 mm Hg, 95% CI −10.7 to −2.6 mm Hg). Eight patients randomized to the conventional therapy group died during the 12 weeks of the study, but none of those assign to treatment with epoprostenol died (*P* < 0.05).

Side effects are common at higher doses of prostacyclin infusion and occur as a result of systemic vasodilation. The most common side effects include facial flushing, headache, and jaw pain, especially when chewing. Other adverse events include diarrhea and bone pain in the legs and bottom of the feet. The side effects were reported more frequently in the group that received epoprostenol. Serious complications included four episodes of nonfatal catheter-related sepsis and one episode of nonfatal catheter related thrombosis resulting in a paradoxical embolism.

Epoprostenol has also been shown to be effective in the treatment of PAH associated with scleroderma. A short-term hemodynamic study of the effect of intravenous epoprostenol in patients with PAH associated with scleroderma demonstrated reductions in pulmonary vascular resistance and an increase in cardiac output [33]. Another single-center, uncontrolled study in patients with connective tissue disease and severe PAH demonstrated the benefits of continuously infused epoprostenol on hemodynamics and exercise capacity [34]. The pivotal trial of continuous epoprostenol for the treatment of scleroderma associated PAH was a randomized, open-label study of 111 patients from 17 referral centers [35]. It compared the effects of epoprostenol as an addition to conventional therapy. More than 90% of the patients in each group were in NYHA functional class III or IV. Importantly, patients with more than mild associated interstitial lung disease were excluded. Over the 12-week study period, the median 6-minute walk distance increased from 270 to 316 meters in the epoprostenol plus conventional therapy group, while it decreased from 240 to 192 meters in the conventional therapy alone group (*P* < 0.001). Hemodynamic assessment demonstrated a decrease in mean PA pressure of 5.0 ± 1 mm Hg in the intervention group, while the conventional therapy arm demonstrated an increase in mean PA pressure of 0.94  ±  1.1 mm Hg (difference between groups of −5.97 mm Hg with 95% CI of −8.98 to −2.96 mm Hg). Cardiac index increased 0.5 ± 0.08 L/min/m^2^ with the addition of epoprostenol, while it decreased 0.1 ± 0.08 L/min/m^2^ in the control arm (difference between groups of 0.60 L/min/m^2^ with 95% CI of 0.39 to 0.81 L/min/m^2^). Adverse events related to the use of a continuous intravenous infusion system for epoprostenol included 4% incidences of sepsis, cellulitis, hemorrhage, and pneumothorax. Similar numbers of patients died in each group, and minor side effects related to the use of epoprostenol were in keeping with other studies.

Epoprostenol's short plasma half-life of approximately 3 minutes mandates continuous intravenous infusion that is both cumbersome for the patient and associated with potentially serious catheter-related complications. Treprostinil is a stable prostacyclin analogue that is chemically stable at room temperature and has a half-life of three to four hours. These properties permit continuous subcutaneous infusion via a needle that can be placed under the skin by the patient. Initial data, published in abstract form, demonstrated the safety and beneficial acute hemodynamic effects of this approach to prostacyclin therapy [36, 37]. The large phase III study that lead to approval of subcutaneous treprostinil for treatment of patients with PAH randomized 470 patients (approximately 80% of whom were in NYHA functional class III) from centers throughout the world [38]. It was the first double-blind, placebo-controlled trial involving patients with PAH. Patients received conventional therapy plus either continuous subcutaneous treprostinil or placebo for 12 weeks. The primary efficacy endpoint in this study was 6-minute walking distance. In the treprostinil group, the median improvement was 10 meters, while the placebo group exhibited no significant change. The difference in median distance walked between the two groups was 16 meters (95% CI, 4.4 to 27.6 meters; *P* = 0.006). While the overall treatment effect was small, patients in the highest quartile dose of treprostinil (>13.8 ng/kg/min) had a placebo adjusted increase in walking distance of 36.1 ± 10 meters. Interestingly, the patients with the lowest baseline 6-minute walking distance in the treprostinil group experienced the greatest benefit. Patients who walked less than 150 meters at baseline experienced a treatment effect of 51 ± 16 meters (*P* = 0.002), while patients who walked more than 351 meters at baseline experienced no substantial treatment effect. These findings suggest that treprostinil infusion may be more effective when used at higher doses in patients with more advanced PAH.

Analysis of cardiopulmonary hemodynamics in this study demonstrated statistically significant improvements in all variables with treprostinil treatment. In particular, cardiac index improved by 0.12 L/min/m^2^ compared to a decrement of 0.06 L/min/m^2^ in the placebo group (*P* = 0.0001 for comparison of change from baseline), and the mean PA pressure decreased 2.3 ± 0.5 mm Hg as compared to an increase of 0.7 ± 0.6 mm Hg in the placebo group (*P* = 0.0003 for comparison of change from baseline). The treprostinil-treated group had a higher incidence of infusion site pain (85% versus 27%) and also had a higher incidence of classic prostacyclin related side effects. Three patients in the treprostinil-treated group experienced an episode of gastrointestinal bleeding. Two of these patients had a supratherapeutic international normalized ratio (INR), and all of the episodes resolved without significant consequences.

Subcutaneous treprostinil infusion allows for the delivery of continuous prostacyclin infusion without the need for a central catheter, and thereby eliminates the danger of central venous catheter infection. At the same time, its longer half-life provides a greater cushion of time for restarting the infusion in the event that it is interrupted because of pump failure or loss of intravenous access. These advantages increase the number of patients who are eligible for continuous prostacyclin infusion therapy. Unfortunately, most patients experience some degree of discomfort at the infusion site, and in approximately a quarter of patients, the degree of discomfort is too great to continue therapy. In addition to subcutaneous infusion, treprostinil can also be used as a continuous intravenous infusion [39].

Prostacyclin analogues also act as effective pulmonary vasodilators when inhaled. Aerosolized prostacyclin was developed to mitigate the incidence of systemic side effects that occur with prostacyclin therapy and to obviate the need for subcutaneous or central venous access and tethering the patient to a battery operated infusion pump. Another potential advantage of inhaled prostacyclin is the improvement of ventilation perfusion matching. This may occur because the greatest pulmonary vasodilator effect occurs in areas of the lung that are well ventilated and thereby reduces blood flow to areas that are not. Several early trials demonstrated the feasibility of this approach in the treatment of PAH. The pivotal trial was performed in Europe and randomized 203 patients with WHO functional class III or IV pulmonary hypertension to inhaled iloprost or placebo [40]. Most of the patients enrolled had WHO group 1 PAH, but nearly 1/3 of patients randomized to iloprost had chronic thromboembolic pulmonary hypertension. Patients received inhalations of 2.5 or 5.0 micrograms of iloprost 6 to 9 times per day as needed. The median inhaled dose in the iloprost group was 30 micrograms per day.

Given their desire for a robust measure of efficacy and the fact that continuous intravenous epoprostenol had previously demonstrated a survival advantage, these investigators chose a combined primary endpoint of an improvement of at least one NYHA functional class and a 10 percent improvement in 6-minute walking distance. This combined endpoint was met by 16.8 percent of the patients who received iloprost as compared with 4.9 percent of the patients who received placebo (*P* = 0.007). There were no significant differences amongst groups with different types of pulmonary hypertension. Adverse effects were similar between the two groups although there was a statistically significant higher incidence of syncope in the treatment group. These events were not associated with clinical deterioration, and the authors concluded that the higher incidence of syncope may have resulted from treated patients increasing their activity too quickly. Given the concern that intermittent inhalations (as opposed to a continuous intravenous infusion of a prostanoid) may not have a durable effect throughout the day, the 12-week hemodynamic assessment included measurement of preinhalation values. While measurements done after iloprost inhalation showed the most improvement from baseline compared to the placebo group, the pre-inhalation change in PVR from baseline of −9 ± 275 dyn·sec·cm^−5^ was still significantly better than the 96 ± 322 dyn·sec·cm^−5^ increase from baseline in the placebo group (*P* < 0.01).

While inhaled iloprost offers a safe and effective form of prostacyclin therapy for PAH without the difficulties of continuous infusion, its short half-life and subsequent requirement for at least 6 inhalations per day makes patient compliance challenging. Each inhalation may take 10–15 minutes to set up and complete. Inhalation of a longer acting prostacyclin may allow for less frequent treatments. Findings from a pilot study of inhaled treprostinil demonstrated that inhaled treprostinil had similar efficacy but a longer duration of action compared to inhaled iloprost [41, 42]. The pivotal trial of inhaled treprostinil for PAH was a multicenter placebo-controlled double-blind study that assessed its' impact in patients already receiving oral therapy with bosentan (125 mg daily) or sildenafil (at least 20 mg three times daily) [43]. Two hundred thirty-five patients with idiopathic or familial PAH, or PAH associated with collagen vascular disease, HIV infection, or anorexigen use were enrolled and studied over 12 weeks. Nearly all patients studied were in NYHA functional class III (98%) at the time of enrollment. They were randomized to inhaled treprostinil (up to 54 micrograms) or inhaled placebo four times daily, and the primary end point was 6-minute walk distance at 12 weeks measured within 10 to 60 minutes after treprostinil inhalation. Secondary end points included functional class, quality-of-life indices and change in N-terminal probrain natriuretic peptide (NT-proBNP) level.

Of the 235 patients randomized, 23 withdrew from the study prematurely. Eight patients withdrew consent, while the rest discontinued due to adverse events or worsening disease. The between treatment median difference in change from baseline in peak six-minute walk distance was 19 meters at week 6 (*P* = 0.0001) and 20 meters at week 12 (*P* = 0.0004). Importantly, there was evidence that benefit was maintained throughout the 6-hour interval between treatments. The median improvement in 6-minute walking distance measured just before inhalation of treprostinil was 14 meters at week 12 (*P* = 0.0066). Amongst the secondary end points, only quality-of-life measures and NT-proBNP demonstrated improvement with inhaled treprostinil compared to placebo.

At the present time 3 different prostacyclin medications are approved for the treatment of PAH in the United States and Europe. No head-to-head studies have been done to compare the efficacy of intravenous or subcutaneous infusion versus intermittent inhalation. However, all appear to be effective to some degree in improving hemodynamics and exercise capacity in PAH. At the present time, clinical trials are being conducted with an orally active treprostinil tablet. These trials may pave the way for the development of a fourth route of prostacyclin administration.

### 5.3. Endothelin Receptor Antagonists

Endothelin was originally identified in 1985 as a vasoconstricting factor isolated from bovine aortic endothelial cells [44]. In 1988, a Japanese group synthesized the peptide and named it endothelin [45]. They noted that it was the “most potent vasoconstrictive peptide so far reported”. Endothelin binds to two different cell surface receptors. ET-A mediates the vasoconstrictor effect of endothelin on vascular smooth muscle. In contrast, ET-B is found predominantly on endothelial cells, where it induces nitric oxide synthase and acts to clear endothelin from the circulation. 

The first endothelin receptor antagonist (ERA) tested for the treatment of PAH was bosentan, which is a nonselective agent that has similar binding affinity for ET-A and ET-B. After a small initial double-blind placebo-controlled study [46] demonstrated improvements in exercise capacity and hemodynamics, a larger study that included multiple dosing regimens was published in 2002 [47]. This study randomized 213 patients with either idiopathic PAH or PAH associated with connective-tissue disease (approximately 90% of whom were in functional class III) to placebo or Bosentan. The bosentan group received 62.5 mg twice daily for four weeks followed by either 125 or 250 mg twice daily for a minimum of twelve weeks. The primary endpoint was the change in 6-minute walking distance.

At the conclusion of sixteen weeks, 6 minute walking distance decreased an average of 8 meters in the placebo group and increased 36 m in the combined bosentan groups (mean difference of 44 meters; *P* < 0.001). There was a nonsignificant trend towards further improvement with the 250 mg dose as compared to 125 mg. Bosentan also significantly increased the time to clinical worsening as compared to placebo. The overall incidence of adverse events between the two groups was similar with the exception of a dose-dependent increase in the incidence of liver transaminase elevation with bosentan therapy.

Ambrisentan is a selective ET-A receptor antagonist that has the theoretical advantage of mitigating vascular smooth muscle induced vasoconstriction without reducing the nitric oxide production and endothelin clearance associated with endothelin's binding to the ET-B receptor. In addition, its longer half-life allows for once daily dosing. The pivotal trials of ambrisentan's use in PAH (ARIES-1 and ARIES-2) were concurrent randomized, placebo-controlled, double-blind studies which differed in terms of the investigative sites and doses of ambrisentan. ARIES-1 randomized patients in North or South America to placebo or 5 or 10 mg per day of ambrisentan. ARIES-2 randomized patients in Europe, the middle east, or Australia to placebo or 2.5 or 5 mg of ambrisentan. In both studies, patients had idiopathic PAH or PAH associated with connective tissue disease, HIV infection, or anorexigen use and the primary endpoint was the change in 6-minute walk distance from baseline to week 12. The results of both studies were published together [48].

At 12 weeks, the six-minute walk distance increased in all ambrisentan groups. The mean placebo-corrected treatment effects were 31 meters (*P* = 0.008) and 51 meters (*P* < 0.001) for the 5 and 10 mg doses, respectively, in ARIES 1 and 32 meters (*P* = 0.022) and 59 meters (*P* < 0.001) for the 2.5 and 5 mg doses in ARIES-2. Improvements in secondary end points such as time to clinical worsening, functional class, symptom assessments, and B-type natriuretic peptide measurements were also seen for the 5 and 10 mg doses. In order to assess the effects of more sustained therapy, these trials also included a long-term study that was open to all patients who completed the initial study and all placebo patients who discontinued because of early escape. Of the 280 patients who completed 48 weeks of ambrisentan monotherapy, the improvement from baseline in 6-minute walk distance was 39 meters.

A third endothelin antagonist, sitaxsentan, demonstrated promise in PAH based on its selectivity for the ET-A receptor, and on a lack of significant interaction with sildenafil. However, the manufacturer withdrew it from all available markets and halted further clinical trials in December 2010 due to problems with hepatotoxicity.

Both bosentan and Ambrisentan have been shown to cause transient elevation of liver transaminases, and these laboratory tests should be monitored at least monthly for as long as the patient is taking them. The ERAs have also been associated with small decreases in hemoglobin and are highly teratogenic. Sexually active patients on these medications should use a double-barrier method of contraception, and fertile woman should be monitored closely for pregnancy.

## 6. Combination Therapy and Treatment Algorithms

Few clinical trials have compared the safety and efficacy of one PAH drug or one class of PAH drugs to another in a head-to-head trial. Wilkins et al. [49] found no difference in 6-minute walking distance in 20 patients treated with the ERA bosentan compared to 20 patients treated with sildenafil for 12 weeks. However, the trial was not powered to detect small differences in functional status or conducted long enough to examine differences in survival. In the absence of comparative trials, treatment algorithms for PAH have been developed using expert opinion [50]. There is general agreement that patients with WHO functional class II and III disease can be treated initially with an oral ERA or PDE inhibitor alone. Inhaled prostacyclins or subcutaneous treprostinil infusion are other options. Those who improve can be kept on their initial therapy. For patients who progress to or present in WHO functional class IV, intravenous epoprostenol is recommended. Other patients who should be considered for intravenous epoprostenol patients include WHO class III patients who have elevated right atrial pressure, decreased cardiac output or other signs of right ventricular failure.

**Figure 3:**
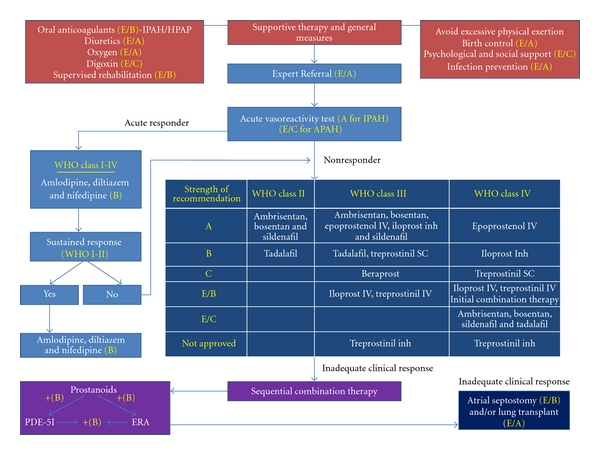
Adapted from [55].

The use of a combination of drugs from different PAH classes is an area of great interest. Several randomized placebo controlled trials have examined the benefit of adding a second PAH medication to patients who failed to respond to or have deteriorated on their initial therapy using a randomized placebo-controlled design [43, 51–53]. The favorable responses described in these trials may be attributed to the combination of the initial therapy plus the add on agent. However, it is also possible that patients in these trials simply responded better to the second agent than the first. Clinical trials that specifically examine the efficacy of a combination of medications to either drug alone have not been completed, although one was recently initiated [54]. At the present time, it is unclear whether patients who do not respond to a given therapy should be switched to another therapy or kept on their original therapy while another therapy is added (see Figure 3). In general, the later approach is more often used due to the fear of deterioration after the original therapy is stopped.

## 7. Follow-Up Care

Continued long-term care is an important aspect of patient survival once diagnosis and PAH therapy has started. Frequent office visit are a necessity for patients starting any continuous infusion or inhaled therapy to ensure accuracy and compliance to therapy. Patients starting on parenteral therapy are seen biweekly to monthly when first beginning therapy. Once a patient's condition has stabilized, they are typically followed in the office every 1–3 months. Along with frequent office visits, 6-minute walk tests, echocardiogram, and plasma BNP levels are performed 2-3 times a year or more often if the patient requires frequent titration of parenteral therapy or addition or change in therapy due to suboptimal treatment response.

Concurrent medications used for PAH patients include diuretics to control peripheral edema as necessary, digoxin, warfarin, and supplemental oxygen. Digoxin may improve right ventricular contractility, but its clinical effect is small [56]. Some studies suggest that survival in PAH is improved with long-term anticoagulation [22], but no adequately powered study has explored this in a randomized placebo-controlled fashion. Unless the patient is at increased risk of bleeding or has other contraindications for long-term anticoagulant therapy, most physicians use low-dose warfarin to target an INR or 1.5 to 2.0 in patients with IPAH. Little data is available to suggest any benefit of anticoagulation in patients with APAH. Oxygen therapy is indicated for patients with saturations below 91%-92% [57] Lower oxygen saturations can increase PA pressures via hypoxic pulmonary vasoconstriction.

Inpatient hospitalization may be required for patient with worsening right heart failure and fluid overload for IV diuresis and stabilization of disease. If a patient's condition does not improve despite an aggressive course of therapy, a repeat right heart catheterization may be warranted to reassess pulmonary hemodynamics.

Referral for lung transplantation should be considered soon after initial diagnosis and initiation of therapy, especially if there is a delay in adequate response to therapy. Lung transplant evaluation should be completed early in the course of therapy, as the evaluation process and wait for suitable donor organ is lengthy, and the patients who fail to respond to medical therapy can deteriorate suddenly. Timing of lung transplant is important in that a patient must be stable enough to tolerate transplant surgery but have a worsening prognosis with a decreased likelihood of surviving without transplant. 

## Figures and Tables

**Table 1:** 4th World Symposium on Pulmonary Hypertension Classification 2008.

(1) Group 1 pulmonary arterial hypertension
(1.1) Idiopathic (IPAH)
(1.2) Heritable
(1.2.1) BMPR2
(1.2.2) AKL1, endoglin (with or without heredity hemorrhagic telangiectasia)
(1.2.3) Unknown
(1.3) Drug and toxin induced
(1.4) Associates with (APAH)
(1.4.1) Connective tissue disease
(1.4.2) Human immunodeficiency virus (HIV) infection
(1.4.3) Portal hypertension
(1.4.4) Congenital heart disease
(1.4.5) Schistosomiasis
(1.4.6) Chronic hemolytic anemia
(1.5) Persistent pulmonary hypertension of the newborn
1′ Pulmonary veno-occulsive disease (PVOD) and/or pulmonary capillary hemangiomatosis
Group 2 Pulmonary hypertension due to left heart disease
(2.1) Systolic dysfunction
(2.2) Diastolic dysfunction
(2.3)Valvular disease
Group 3 Pulmonary hypertension due to lung disease and/or hypoxia
(3.1) Chronic obstructive pulmonary disease
(3.2) Interstitial lung disease
(3.3) Other pulmonary diseases with mixed restrictive and obstructive pattern
(3.4) Sleep disordered breathing
(3.5) Alveolar hypoventilation disorders
(3.6) Chronic exposure to high altitudes
(3.7) Developmental abnormalities
Group 4 Chronic thromboembolic pulmonary hypertension
Group 5 pulmonary hypertension due to unclear multifactorial mechanisms
(5.1) Hematological disorders: myeloproliferative disorders, splenectomy
(5.2) Systemic disorders: sarcoidosis, pulmonary langerhans cell histiocytosis, lymphangioleiomyomatosis, neurofibromatosis, vasculitis.
(5.3) Metabolic disorders: glycogen storage disease, Gaucher disease, thyroid disorders.
(5.4) Other: tumor obstruction, fibrosing mediastinitis, chronic renal failure on dialysis.

**Table 2:** World Health Organization Functional Class of PAH.

Class I: Patients with PAH that causes no limitations on physical activities. Routine physical activity does not cause increased dyspnea, chest pain, fatigue, or presyncope.

Class II: Patients with PAH that causes mild limitations on physical activities. Patients are comfortable at rest but routine physical activity results in increased dyspnea, chest pain, fatigue, or syncope.

Class III: Patients with PAH that have marked limitations on physical activities. Patients are comfortable at rest, but less than routine physical activity results in dyspnea, chest pain, fatigue, or palpitations.

Class IV: Patient with PAH that results in the inability to perform any physical activity without symptoms. These patients may have signs of right heart failure. Dyspnea with or without fatigue may be present at rest, and symptoms are increased by any physical activity.
